# Transition processes for newly qualified paramedics entering primary care: a critical discussion and theoretical perspective

**DOI:** 10.3399/bjgp24X737337

**Published:** 2024-04-26

**Authors:** Joe Copson, Georgette Eaton, Kamal R Mahtani

**Affiliations:** School of Health and Social Work, University of Hertfordshire, Hatfield; Lecturer, Paramedic Science, School of Health Sciences, University of East Anglia, Norwich.; DPhil Evidence Based Health Care, Nuffield Department of Primary Care Health Sciences, University of Oxford, Oxford.; Nuffield Department of Primary Care Health Sciences, University of Oxford, Oxford.

## Background

Paramedics within the first 2 years of registration are referred to as ‘newly qualified paramedics’ (NQPs). The most recent survey of the paramedic workforce in primary care found that 7.6% (*n* = 26) of responders had been a paramedic for less than 2 years prior to moving into their primary care role.^1^ This figure suggests that a proportion of NQPs are seeking employment opportunities outside the traditional ambulance service employer, despite the assumption that paramedics working in primary care should possess a minimum of 3–5 years of experience.^2^ Indeed, the number of paramedics employed in primary care has seen a remarkable uptick since 2016,^3^ though this is not surprising given that paramedics have been specifically emphasised as a profession for recruitment into primary care.^4^ This is an emphasis supported in particular by specific funding models in England.^5^

The appetite for paramedics to fulfil workforce gaps existing in primary care is not surprising — educated to degree level with autonomous registration, they are inherently versatile and function as generalists, dealing with the undifferentiated caseloads that characterise the 999-emergency service. It is this latter point that gives the profession its ‘USP’ to work in primary care, and recent work on this subject has indicated that it is the clinical experiences paramedics have in ambulance services that give them the clinical foundation to work in primary care settings.^1^^,^^6^ However, questions arise about the opportunity cost to ambulance services of experienced paramedics transitioning to primary care, and the suitability of employing paramedics in primary care who have only recently obtained their registration.

## Education and experience

The nature of ambulance calls has become increasingly diverse, necessitating paramedics to evaluate, treat, and formulate ongoing care plans for an assorted patient population. In the 12 months to December 2023, just 11% of 999 calls were initially triaged as immediately life threatening, and nearly half (48%) of 999 calls did not result in conveyance to an Emergency Department.^7^ Immersed in this complexity, NQPs are expected to exercise independent clinical practice as the sole registered clinician on an ambulance from the beginning of their career.

Paramedic degree programmes are regulated by the Health and Care Professions Council and may receive endorsement from the College of Paramedics.^8^ Undergraduate paramedic education must address diverse patient scenarios in challenging environments, posing curriculum design challenges for time-limited courses. For example, despite the relatively low frequency of advanced life support in ambulance calls, its importance cannot be understated. Programmes cover extensive content on patient assessment, anatomy, pathophysiology, and pharmacology. While some programmes include placements in primary care settings, this is not mandatory. When such a placement is offered, student paramedics may gain exposure to the primary care environment, but without the responsibility of autonomous practice.

Within ambulance services, NQPs follow a structured 2-year programme to support their transition from student to autonomous professional, with a portfolio that enables NQPs to demonstrate the development of their academic knowledge, skills, and clinical experiences into confident practice.^9^ In some ways, this is not too dissimilar to the foundation years of medicine. However, increasingly NQPs may seek alternative employment after less than 2 years in an ambulance role, or even directly from university, meaning such a portfolio is obsolete.

The motivations for paramedics seeking alternative employment are likely to be varied.^10^^,^^11^ Research exploring paramedic experiences during transition to primary care suggests improved work–life balance, increased autonomy, and new challenges are motivating factors.^12^ However, motivations such as a desire for new challenges may be more common among experienced paramedics than NQPs. Multiple factors including individuals’ intrinsic motivations to join the profession, the move to a degree-level threshold of entry, ambulance service work environments, or other considerations such as medical exclusion from obtaining driving licences may influence the motivation of NQPs entering primary care.^13^^–^^15^ Understanding the experiences of NQPs during transition to the workforce is imperative for ensuring patient safety and staff wellbeing. Within the ambulance service context research is ongoing in this space.^16^ Given the presence of NQPs working in primary care, and the continuing development of the paramedic profession, we argue that careful attention should be paid to the transition process for NQPs assuming roles in primary care.

## Theoretical models of transition

Facilitating successful transitions is challenging because of complex sociological, psychological, and environmental factors. Understanding, and applying, theoretical models of transition can be a helpful way to conceptualise how NQPs may experience role transition. First, we use Schlossberg’s Transition Model^17^^,^^18^ to consider the types of transitions that NQPs may be experiencing when commencing primary care careers. Second, Bridges’ Transition Model is applied to NQP transition to primary care, providing an overview of considerations during a staged transition process.^19^ Though our application of these models is focused on NQPs, the theoretical concepts and considerations for practice could be of equal utility if applied, with appropriate sensitivity to context, to the transition of various other clinicians who may enter primary care.

### Schlossberg’s Transition Model

The motivations driving NQPs’ transition may be multiplex, and expanding opportunities for employment within primary care may result in relatively sudden changes in career trajectories. Schlossberg outlines three main transition types.^18^ In the context of NQPs working in primary care, we may consider the following:

#### Anticipated transitions

Some NQPs may have chosen to transition from the ambulance service to primary care as part of their career plan. They have anticipated this change and may have prepared for it through their undergraduate education, or in the first formative months of their NQP role.

#### Unanticipated transitions

Others may have been thrust into primary care unexpectedly because of circumstances such as medical issues, changes in employment, or organisational restructuring. A transition of this type is perhaps more likely for NQPs, who arrive in primary care having had less time to align the intrinsic motivations that inspired them to join the profession, with a role in primary care. NQPs under such a transition may therefore face challenges in adapting to this unexpected transition.

#### Non-events

Some NQPs may have had different expectations about their career transition. For instance, they might have initially planned to continue working, and progressing, in the ambulance service for a more extended period but ended up transitioning to primary care earlier than they had foreseen.

### Bridges’ Transition Model

Bridges’ Transition Model focuses on the psychological and emotional aspects of change and transition, and outlines three phases of transition: the ending phase, the neutral zone, and the new beginning.^19^ Overall, this model emphasises the need for support, learning, and self-reflection, which can be particularly relevant for NQPs as they navigate the shift from being a student in the ambulance service to working in primary care (outlined in Box 1).

**Box 1.** Bridges’ transition model^19^ applied to NQPs transitioning to primary care

Ending phase	Transition catalysts	Understanding transition catalysts, such as career aspirations, job opportunities, organisational changes, or personal life circumstances, can assist NQPs in navigating new roles and enable employers to provide effective support
	Letting go of the old role	NQPs undergo a symbolic departure from the ambulance service, breaking with their past experiences during their training. This phase includes recognising the end of their ambulance service career, accepting the shift to a new environment, and adapting their paramedic identity to new role expectations in primary care. This transition affects NQPs’ identity, self-esteem, and professional perception. They may utilise personal resources, such as skills and knowledge, and seek external support from peers, mentors, colleagues, or professional development opportunities to navigate this change. During this phase, the effects of the deconstruction of university support networks must be considered, as peers and previous mentors likely remain in the ambulance service context
	Navigating ambiguity	Moving from a clearly understood paramedic role in an ambulance service to a less well-defined primary care role can cause disorientation, uncertainty, and ambiguity for NQPs
Neutral zone	Transition state	This adjustment phase involves learning new skills, protocols, and procedures while letting go of previous practices. NQPs may grapple with identity conflict, questioning their status as paramedics and how their background informs their primary care role
	Seeking support and learning	NQPs may seek guidance from mentors and colleagues to acquire the knowledge and skills for their new roles, marking a time of experimentation and discovery. Availability of support systems, both within the workplace and in their personal lives, is crucial for NQPs’ transition, with effective mentorship, peer support, and guidance enhancing the adaptation process. Ideally, NQPs would follow a predetermined sequence of actions in this stage, with roles and procedures clearly established. However, in primary care, this may differ, and NQPs might need to align with other healthcare professionals, working under their guidance to gain authority. It is vital for supervising clinicians to recognise the limited autonomous clinical experience NQPs bring
	Managing emotions	NQPs may go through a range of emotions, including anxiety, frustration, and excitement, as they navigate the challenges and opportunities of their transition. Recognising and managing these emotions is important for a successful adjustment
New beginning	Integration and embracing the new identity	In the final phase, NQPs integrate seamlessly into the primary care team, contributing unique experiences, enhancing patient care and collaborating effectively with colleagues. NQPs fully embrace their roles in primary care, forming a hybrid professional identity as healthcare providers, navigating their identity by blending paramedic skills with primary care norms and practices, offering a unique perspective in patient care. Role identity evolves with experience, and NQPs may refine it further through education or training aligned with their evolving responsibilities. With increasing experience, they become more committed to their identity as primary care professionals, gaining a deeper understanding of their role’s significance
	Building confidence	As NQPs gain experience and competence in primary care, confidence and self-assurance grow and they become more comfortable and effective in their new roles
	Continuous learning	The transition is an ongoing process, and NQPs should recognise the importance of continuous learning and adaptation as they further develop their careers in primary care

*NQP = newly qualified paramedic.*

## Practical implications: supporting the NQP during transition

**Figure 1.**
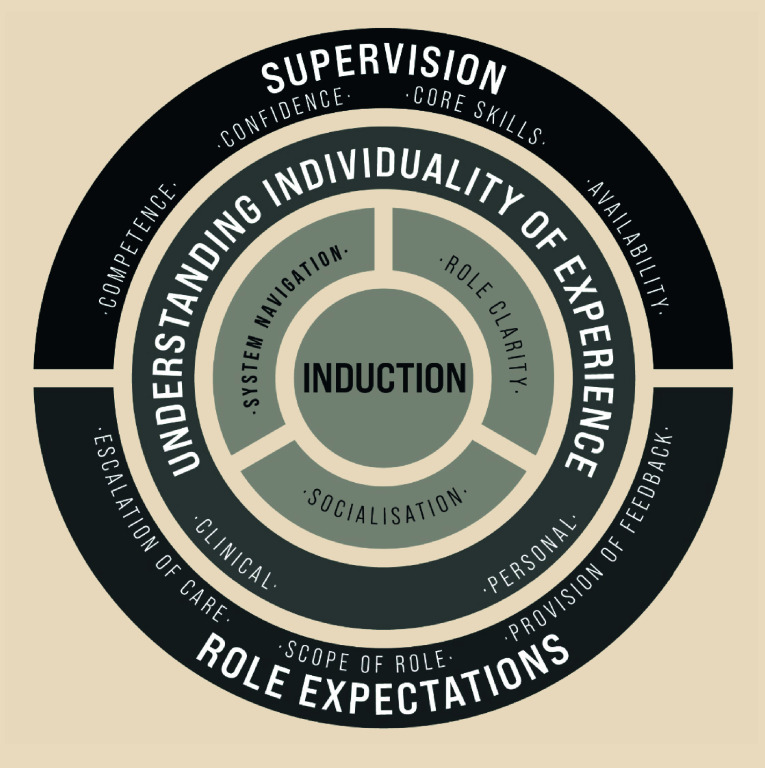
A model to support NQP transition to primary care. NQP = newly qualified paramedic. Adapted from: Eaton G. Using realist approaches to explain and understand the optimal use of paramedics in primary care.^20^

Based on the substantive theories of transition, and understanding of transition processes, we make recommendations, and highlight considerations, for supporting the NQPs’ transition to primary care (outlined in Figure 1). Effective support during transition is essential for patient safety and NQP wellbeing, and is likely to increase recruitment and retention of staff.

There may be a paradoxical relationship between the desire of the NQP for direct supervision and support, and the desire for autonomous practice and establishing their professional identity. It is important to consider that the NQP is transitioning from a structured educational programme with supervised placements, formal feedback, and academic support. The practical implications of the transition to primary care practice may require supporting the NQP in multiple areas and from varying perspectives.

### Induction

A thorough induction to the workplace is essential. Inductions should be focused, including but not limited to expectations of the NQP, navigating systems and locations, clarity around support and supervision availability, and introductions to supervisors and socialisation with the wider multidisciplinary team.

### Understanding individuality of experience

Understanding the individual’s ‘situation’ through initial conversations can help to support transition. Some areas to focus on may be the factors that precipitated the transition into primary care, motivations for wishing to work in primary care, concurrent stressors or external situations, and local links/support network.^18^

External support systems are particularly important to establish, as previous formal support systems at university, or through previous mentors, may not be available. NQPs may be moving away from family to commence their careers, and it is likely that peers from university will be transitioning into paramedic roles other than primary care, and in different regions of the country. The potential unravelling of support systems at this stage must be considered.^18^

Understanding individual experiences prior to joining primary care is important to support transition. Individuals may have a variety of life and work experiences that will shape them as a clinician and influence their transition to primary care.

### Supervision

Effective supervision is crucial during the initial transition to practice. The supervisor’s role should be clearly articulated to the NQP. It is important that supervision is readily available, and an appropriately trained, named individual is responsible for overseeing transition.

It is the responsibility of the practice, at the outset, to delineate how core skills will be assessed, and to ensure that the NQP is competent and confident in performing these clinical skills before allowing them to practise independently.

Supervisors should regularly review the transition with the NQP, as the NQP themselves may initially struggle to identify exactly where they are within their transition from an emotional, psychological, and clinical perspective. Self-reflection, without formal feedback mechanisms, can take time to develop.

Providing such support to an NQP is clearly a resource-intensive undertaking. To ensure that employers can effectively support NQPs transitioning into primary care, and other clinical environments, commissioning bodies should consider transition to practice requirements as part of their funding allocations to practices.

### Role expectations

Delineating the role of the NQP during transition is essential, and this may be facilitated using a staged approach. The role of the NQP may change as they gain experience and have been assessed as competent in various situations.

It is important to discuss the initial expectations, and the incremental stages of transition, from the outset. Particularly when transitioning to an environment in which they possess less experience, clarity around expectations and scope of practice, and when to seek support, are fundamental to ensuring patient safety.

The ability of the novice clinician to manage their own bandwidth, and clinical complexity, will be varied. It is likely this ability will be reduced relative to an experienced clinician. Considering these phenomena is imperative; some practical examples may be: beginning with direct supervision through all consultations, seeing a smaller variety of presentations, and having extended appointment times.

## Conclusion

Transition to professional practice is a labyrinthine process for any newly qualified health professional. The path of transition is rarely unidirectional, and the NQP transitioning into a primary care role may face additional challenges including contextual unfamiliarity, identity, support networks, and role expectations. In this article, we have provided an outline of the potential transitional considerations within a theoretical perspective, to encourage reflection and discourse on how best to facilitate these transitions. Although our discussion centred on NQPs transitioning to primary care, the principles and theoretical foundations discussed in this article can be effectively applied to various clinical backgrounds when contemplating the transition to primary care practice. To successfully support the transition of an NQP into primary care, employers must take an individualised approach, providing tailored support and recognising the challenges of transition, as well as empowering NQPs to utilise the unique skillset and experiences that individuals bring to the role. To facilitate this, funding bodies may consider providing financial support for practices to properly embed and support newly registered clinicians, recognising the supervisory premium required. Such funding should be ringfenced specifically for supporting new clinicians through transition to the practice environment, with allocated time each week for tutorials, supervision, and educational development.
